# Academy of Stomatology

**Published:** 1905-07

**Authors:** 

**Affiliations:** Academy of Stomatology


### ACADEMY OF STOMATOLOGY.

A REGULAR meeting of the Philadelphia Academy of Stomatology was held at its rooms, 1731 Chestnut Street, on the evening of Tuesday, February 28, 1905, the President, Dr. I. N. Broomell, in the chair. A paper was read by Dr. Hart J. Goslee, of Chicago, entitled "The Rationale of Pulp Extirpation."

(For Dr. Goslee's paper, see page 228.)

### DISCUSSION.

*Dr. R. Ottolengui*, New York City.—There is very little in the paper with which I feel inclined to take any exception, and if you will pardon me I will direct my remarks to the general subject rather than to the paper. It is a remarkable fact how little we know about pulp removal, one of the most important operations the dentist is called upon to perform. There are two methods by which operations become established. Usually we have a period of experimentation followed by a record of what may be called clinical experiences, dental deductions which may be known as clinical facts, and it is only at the very end that the real scientist comes in and sifts these clinical facts in order to find the scientific truth. The paper before us is admitted by the author to be a contribution to the knowledge of clinical facts, a compilation of deductions from experience. A great many dental topics have been made almost a life study by our scientists, but little of real science has been gathered on this question of pulp extirpation. What do we really know about the series of results from this application of arsenic? We tell our patients, many of us, "I am obliged to kill the nerve." As a matter of fact, we do not even try to kill the nerve, but we try with our arsenic to so act upon the pulp as to make removal possible without pain. There is the difference between the assertion and the actual fact. Has it actually been demonstrated beyond scientific argument just why the application of arsenic enables us to remove the pulp without pain? I can scarcely believe it is the action of the arsenic upon the nerve-fibres that allows this, because we have all, perhaps, had experiences in which pulps were painful after many applications of arsenic. I may be wrong, but I think that the general explanation is that the arsenic acts as a constringent of the capillaries.

Just how this constringent effect enables us to painlessly remove the pulps is a question. If we wait until this disorganized tissue separates at the apex, then, of course, we have dead matter which is removed painlessly. Apparently, however, we usually remove that pulp not painlessly, but with less pain, after the application of arsenic than in its normal condition. We are all clamoring for an established mode of procedure. The application of arsenic as a means of painlessly removing pulps has been an established procedure for half a century, with little complaint against the method. Now we have a new method offered, but I call your attention to the fact that the new method is even now, five or ten years after introduction, rather in an empirical state. How much of actual study has been given to the desensitizing of pulp tissue by cocaine pressure? Who of us knows exactly what happens when cocaine is applied to the pulp, and that after the application of the cocaine and of pressure, this tissue can be removed painlessly? Gentlemen, I ask you again, who knows? Who has tried to find out? Here I meet my own dilemma. I have tried to find out, but I have not the histological and chemical knowledge with which to solve the problem, but you have in this scientific centre of research a number of gentlemen who, I think, have the equipment; and if you will pardon my taking the time, I will outline a mode of clinically studying this problem with the hope that Dr. Broomell or Dr. Truman, or one of the many others with you, may take this up. Something more than a year ago I began the microscopic examination of pulps which I removed. I noticed an extreme difference between the pulps treated by arsenic and those by cocaine. It must be remembered that in the pulps treated by arsenic there is a constringence of the capillaries with the compulsory retention of a certain proportion of blood within the capillaries, and the pulp removed is a brilliant red. If you remove the pulp-tissue while the capillaries are still engorged with blood, you leave all the blood that was in the pulp in the canal and bring forth a whitened tissue. By allowing these removed pulps to dry it has seemed to me that I have been able to detect crystals, which I have taken to be crystals of arsenic. In the pulps treated by cocaine I have also seen crystals which are of a different appearance. This should be a question for the scientists, and if it be a fact that we can thus make visible the crystals of the drug used, it should be established.

The next fact to be determined is whether, if we can force these medicines along the capillary tracts, they will pass beyond the apex. I suggest the following experiments, which I had hoped to have carried out in New York, but for which I have lacked material, having seen but three freshly exposed pulps this winter. My plan was to determine the action of the cocaine or arsenic, and also the presence or absence of bacteria. First, upon exposing the pulp I would collect some of the blood and examine it for the bacteria, and keep it for comparison in the future. Secondly, my plan would be to cocainize this pulp, remove it, and make a slide to be examined under the microscope or by chemical means, to determine whether the cocaine actually passes into the pulp-tissue, and if so, whether it follows the blood tracts. Finally, upon removal of the pulp, collect the first blood, the blood which was originally in the pulp, and discover, if possible, whether the blood contains cocaine or bacteria. This would aid in deciding whether or not we have forced bacteria into the pulp in applying the cocaine. By another series of experiments we might first disinfect or sterilize the cavity, when, if bacteria were found in the blood of the pulp after its removal, we would know that the infection had been in the pulp prior to its removal. If it is possible for an exposed pulp to absorb chemical agents, why should it not suffer from ptomaine poisoning when in contact with caries said to be of bacterial origin? If it be proved that there may be bacteria in the pulp where there is deep-seated caries, and if it be proved that by pressure we can force chemicals and bacteria beyond the apex, we may in this way account for lame teeth after the treatment by cocaine pressure. My final experiment would be to allow all of this first lot of blood to pass away, the canal to be several times washed with sterilized warm fluids, and then to make several examinations of the blood coming from beyond the apex, to determine the presence of bacteria and cocaine. We need the aid of scientific research along these lines to know beyond peradventure just what we are doing. I hope some one may be interested to take up the work.

*Dr. F. J. Capon*, Toronto.—I feel that I am somewhat under false colors in being placed on the programme to discuss this paper, as I am merely a visitor in your city.

I must congratulate Dr. Goslee on the splendid essay he has presented, and also upon the practical way in which he has set the subject before us.

I may say that I have almost entirely adopted the pressure anæsthesia method for the extirpation of pulps, either by dealing with the exposed pulps directly, or by way of the dentinal tubules, and find each of them very efficient in their indicated place. One with a busy practice will naturally drift away from the difficult to an easier way of doing an operation, if the results obtained are as satisfactory. I was one of those who in the year 1894 or 1895 paid twenty-five dollars to Dr. Funk, of Chicago, to learn this, a secret method at that time, and I have employed it more or less ever since, and have found it so satisfactory that the arsenical method has been almost cast aside.

In those cases where there is a deposit of dentine in the pulp-chamber I prefer to take the chances with cocaine and pressure rather than with arsenic, but they are often troublesome with either. This again may be said when teeth are badly decayed below the gum margin and an aching and exposed pulp exists. Pressure anæsthesia is then especially indicated as a speedy and safe treatment.

The after-trouble that has been spoken of is not more frequent with immediate extirpation, although a soreness is more liable when bleeding has not followed the removal of the pulp. I must be fortunate with my treatment, as I do not experience the soreness that has been mentioned to-night. When dealing with an anæsthetized live pulp a sterilized broach should be used.

*Dr. Emory A. Bryant.*—As a usual thing, I am not in the habit of agreeing with a man simply to "pat him on the back," as is a generally observed custom in the discussions of papers read before dental societies, but I must confess that the ideas as expressed by the essayist this evening so closely coincide with my practical experience with pulp extirpation that I am at a loss as to what to say.

I cannot, however, resist the temptation of referring to that part of the paper quoting my friend B. Holly Smith in regard to the ill-effects of the use of arsenic in pulp extirpation upon the tissue as well as the teeth. I doubt very much his ability to prove his assertions, or that any such results have to be contended with by careful operators.

From the paper one would judge that in 1904 Dr. Smith discovered an unknown characteristic property of arsenic in its dental uses, but in referring to a card published to the public in April, 1881, by Dr. Williams Donnally we find the following:

"In treating the natural teeth my object is to preserve every one that time, patience, and modern methods of treatment can save. The use of arsenic in destroying exposed pulps is condemned by the more enlightened of the dental profession, on account of its turning the teeth dark, inducing abscess, and, finally, the loss of the teeth. The pulp, which is the source of life to the tooth, should always be treated, when exposed, with a view to its restoration to health."

Thus, you see, Dr. Smith's discovery was previously published to the world by his friend Dr. Donnally by some twenty-three years, and if it was a fact at that period, it would seem strange that the profession has so long failed to recognize the disastrous effects, and only awake to realization upon the advent of pressure anæsthesia as a substitute.

I was so struck with the force of Dr. Donnally's argument that I published it in an article of my own in the June, 1903, *Items of Interest.*

Pressure anæsthesia is one of the greatest aids to quick work under certain conditions that has been given to the profession. Those conditions are laid down so perfectly by Dr. Goslee that it is useless to further refer to them. Practically speaking, I cannot say that I have had any of the ill effects spoken of. I had the pleasure of seeing, the other day, a patent, taken out seventeen years ago, upon an instrument which is practically the pressure anæsthesia instrument of to-day. My friend used in this phenol sodique, carbolic acid, or alcohol, and, as he stated in a letter which I received from him the other day, he had been using pressure anæsthesia for years and did not know it. He could not remember at what date he commenced the use of cocaine, and in that date I am much interested, inasmuch as there is now before the Patent Office an application for a patent upon the method and process of producing pressure anæsthesia, and unless that application can be antedated by the publication of the fact of the use of pressure anæsthesia previous to the application for a patent, the profession is liable to again have to contend with a state of affairs similar to that it had with the Goodyear Dental Vulcanite Company and the International Tooth Crown Company.

If that patent is once allowed, the profession will have to go into the courts to prove its previous use, and I hope to have the necessary data to place before the Patent-Office in the near future, so that the patent will not be issued. I wish to thank Dr. Goslee for his timely paper, and the members of this society for so patiently listening to me.

*Dr. Charles F. Ash,* Brooklyn.—I have been much interested in the subject of pulp extirpation. The trouble seems to be not in getting the pulp out, but in the results which ensue in some cases. Eleven years ago there came to my office a case of exposed pulp so intensely hypertrophied that it was impossible for the patient to use that side of the mouth. He had had the tooth treated, but without success. It was exceedingly sensitive, and I made on that occasion my first application of pressure anæsthesia. The thought was original to me then, and I used pressure anæsthesia with cocaine and a piece of unvulcanized rubber with good success. I removed the pulp in not more than fifteen minutes. I treated the root, and the patient returned in a few days, when I put a filling of gutta-percha in the root and of cement in the cavity. That tooth is still doing good service, and has never given any trouble. From that time on I have used pressure anæsthesia in that way. It seems to me that the gist of Dr. Goslee's paper is contained in his statement that success in pulp extirpation lies in the judicious selection of the kind of treatment for a given case. I did not cease using arsenic when I discovered that I could use pressure anæsthesia, nor have I stopped using it to this day. I have found cases in which I was able to successfully use pressure anæsthesia and remove the pulp from two canals, while the other canal was too small for the smallest broach, and in this I have applied arsenic. These are the cases in which a good many fail because they do not carry their efforts far enough. I have not had a case of pressure anæsthesia followed by continued soreness or lameness, as stated by some gentlemen. I have had cases in which there has been a temporary soreness, but I think this has been due to a slight secondary hemorrhage occurring after the effect of the adrenalin chloride had ceased. Until that blood is absorbed there is apt to be soreness.

*Dr. Frank Bliven,* Worcester, Mass.—I thank you for the invitation to discuss this subject. We all appreciate the paper of Dr. Goslee and the able way in which he has presented it. Dr. Ottolengui has entered into the scientific side of the question and given us his admirable views. We have also listened to other men, and it is a question whether there remains much to be said. Dr. Ottolengui has made a statement relative to the dead matter left in the root. There is no such thing as dead matter. If the matter were dead and left in the root it would be inert and could not possibly cause future trouble. It is live matter left in the root, and it depends upon the character of that life what the result will be. Matter may change indefinitely in its form, but we cannot eliminate it from the universe. It was my pleasure to listen, in New Hampshire, to quite an able paper on this subject and to the discussion upon it. There was an old man there with a great deal of experience, but with little scientific knowledge, and, after all the scientists had got through with the discussion, he rose and said,—I will try to repeat his words as spoken,—"Well, you scientific men know a lot about this killing pulps and taking them out and filling up the canals and performing scientific operations, and getting a big fee for them; but when I commenced practice we did not have time for that sort of thing. All we used to do was to put the arsenic into the tooth, and when we thought the nerve was dead we took out what we could get out and put in cotton and creosote and filled up the cavity, and we did not have any more trouble than you do now." There is a lot of truth in what he said, for they did not have much trouble. But the scientific side is now before the profession, and it calls upon the ablest men we have to solve the problem and ascertain whether we do force what Dr. Ottolengui has called dead matter, or what we call live matter, beyond the apical foramen. I think much depends upon our judgment of the application best suited to the individual case. When we have had long experience with pressure anæsthesia we shall perhaps be able to form better deductions from our results. After all possible scientific light has been brought to bear upon this subject, I still believe that it will depend wholly and entirely upon the condition or personal equation of our patients what the result will be.

*Dr. Ottolengui.*—I did not say anything about pressing dead matter through the apical foramen. If a pulp treated with arsenic were left in the mouth long, so that it separated from the parts beyond, it might be called dead matter, and therefore its removal would be painless. We know that what we call infection is from something quite alive, since it is capable of reproduction.

*Dr. Joseph Head.*—Dr. Goslee has referred to the possibility of the dental pulp absorbing the arsenic which might be carried through the tissues. Dr. Flagg in his experiments took a number of dental pulps and, after snipping off the portion that had been touched by the arsenic, applied Marsh's test, and found no arsenic present. He therefore felt that the pulp was not destroyed by arsenic poison, but by a peculiar arsenical impress, which is not understood, except that it caused strangulation of the pulp at the apical foramen.

Dr. Ottolengui brought out the point that we did not have scientific knowledge of the way in which a pulp was affected by the cocaine. We know that when cocaine is injected into the tissues it has a certain well-defined physiological action, and that the cocaine has an effect on the terminals of the nerves. Cocaine almost to a certainty acts on the pulp in this way. By application of the cocaine to the perfectly healthy nerve, and with sufficient pressure to force a little of it into the tissue, the circulation, where there is circulation, in half a minute will give us anæsthesia. We also know that where there are pulp-stones, and where the circulation has been lessened, we have greater difficulty. Pulp anæsthesia as the result of the cocaine injection seems to me a simple problem.

*Dr. J. H. Gaskill.*—I think we can get as complete anæsthesia of the pulp without the use of cocaine as we can with it. I have frequently produced anæsthesia with carbolic acid, and I question whether it is really the cocaine which produces the anæsthesia.

*Dr. G. E. Root.*—I believe that we can get pressure anæsthesia when using Schuylkill water as well as when using a solution of cocaine. I believe that it is the pressure which produces anæsthesia as much as the cocaine, and with the use of the arsenic I think the anæsthesia is not so much the result of the action of the arsenic itself as that the arsenic causes a contraction at the apex of the pulp and we have the anæsthesia from the congestion rather than from the arsenic.

*Dr. James Truman.*—I am particularly gratified to find that one of the younger members of our profession has so far sustained the verdict of fifty or more years, that arsenic is worthy of preservation. We have heard much in recent times and through our journals from those who assert that they no longer use arsenic, and that pressure anæsthesia is the one thing for the present and for the future in the eradication of pulps. I have had some experience with the latter, and I have had some fifty years' experience with the former. I do not understand why it is that men in practice who make use of arsenic condemn it. My only explanation is that the general profession, as a rule, do not understand the action of arsenic and do not fully realize the action of cocaine. The use of arsenic in the past has been largely empirical, and it is empirical to-day. I find the majority using arsenic without any clear idea of the amount that is necessary to destroy the pulp of a single-rooted tooth, and it is no wonder that it frequently is carried beyond the pulp to produce periodontitis. I try to impress upon my students that we must get away from the old method, introduced by Dr. J. D. White, of applying arsenic without any scientific basis.

It was demonstrated by Miller, in his celebrated experiments upon mice, that arsenic was a paralyzer, and all my own clinical experience has equally demonstrated that its effect is to paralyze the sensory nerves, cutting off nutrition. Cocaine does precisely the same thing,—paralyzes the sensory nerves. That has been understood as one of the peculiar properties of this agent, and why should we here to-night hear the question asked, "How does cocaine act?" It is very well known that in the injection hypodermically along the margin of the gums sloughing is frequently produced. The explanation of this may be found in the local disturbance of the vasomotor system, cutting off nutrition. I believe this to be scientifically correct. The pressure method has the same antagonism to meet as arsenic, in that where there has been long continued inflammation we find that arsenic and cocaine are equally antagonized by the congested vessels of the pulp. We have to-day to meet each case separately and judge accordingly. I would not contend that either method is absolute; we may probably never get away from arsenic, and we may find in the future that all the difficulties that have been enumerated by the essayist will be found increasing in proportion as we make use of the so-called pressure anæsthesia.

*Dr. Edwin T. Darby.*—I think I voice the sentiment of the Academy when I say that it has listened with the greatest pleasure to Dr. Goslee's paper as well as to the discussion which has followed. I thought while Dr. Goslee was reading his paper that if I could write with the same apparent ease that he does, and deliver it with the same grace that he did, that I should write oftener for the Academy.

The point concerning the danger of infecting the pulp and the tissue beyond by forcing septic matter into the pulp-canal was new to me. It may be remembered by some of you that at the meeting of the National Dental Association held at Old Point Comfort, some years ago, before the pressure method of anæsthetizing pulps was introduced, I narrated an experience that I had had in anæsthetizing a pulp by injecting with a syringe a solution of chloroform and carbolic acid. I practised this method for some time with the greatest satisfaction. The first case was that of a lateral incisor which I was preparing for a filling. The cavity was confluent with one on the opposite side, and so frail was the tooth that I broke the crown near the gum. My patient was to sail for Europe the next day, and it gave me no little concern. I took a small hypodermic syringe, and with a canula instead of a needle inserted it into the pulp-chamber (first laying a crystal of carbolic acid upon the pulp to paralyze it). I injected chloroform and carbolic acid into the pulp-canal, and in less than half a minute I had anæsthetized it, and with my brooch took it out, prepared the canal, and put an artificial crown upon the root. I knew nothing then of using cocaine. In a case at the University, where I sought to demonstrate this method, I broke the canula of my syringe, but substituted a pellet of bibulous paper well saturated with carbolic acid and chloroform, put it into the cavity of decay, placed some soft rubber over that, and forced the carbolic acid and chloroform into the pulp and extracted it in less than a minute.

I have heard men say that the anæsthetization can be produced as well with water, chloroform, alcohol, or other fluids as with cocaine. I have not experimented with these, but I do know that by the use of cocaine crystals I have brought about the desired effect. I do not understand what the gentlemen mean by making a solution of cocaine for pressure anæsthesia. My own method is to put the cocaine crystal into the cavity and my rubber immediately upon that, and by the time I am ready to make pressure the moisture from the air and the moisture in the tooth has given me a deliquesced solution of cocaine. The mistake is often made of trying to use a solution of cocaine of insufficient strength. I see no harm in using the crystals direct, even though you use more than enough to anæsthetize several pulps. The chances are that you will not force it beyond the foramen of the tooth.

*Dr. Ottolengui.*—I desire to make reply to some of the remarks that have been made. I am the one who expressed the view that we know little about the physiological action of cocaine or of arsenic in the pulp. I am glad to have had my attention called to Dr. Miller's paper, but unless Dr. Miller tells us much more than

Dr. Truman has told us, I fail to see how we are any nearer to the facts that I am trying to discover. Dr. Truman says that arsenic paralyzes the nerves. If we say it anæsthetizes the nerves, we know almost as much as when we hear that it paralyzes. The paralysis of the nerve is the making of the nerve inert. If the nerve is inert and ceases to act, the resultant lack of muscular effort which that nerve is supposed to supply is called paralysis. In sensory nerves, if we have inaction we have lack of sensation. We have the condition, but are not told what produces it. Dr. Head has said that just exactly what anæsthesia is we do not know. It would require a great many experiments similar to those of Dr. Flagg to determine this. We believe that the presence of pulp-stones hinders the action or result that we hope to obtain, and I know that in pulps which I removed under cocaine anæsthesia, when the pulp-stones were present there was this appearance of crystals all around the pulp-nodules, with no such appearance beyond these pulp-nodules. This carries out my view that cocaine actually enters the pulp-tissue; if it does not enter the pulp-tissue it cannot be carried beyond. That is a point which has not yet been determined.

I ask any gentleman to show me the expression "pressure anæsthesia" anywhere in our literature before it was used by Dr. W. J. Morton.

Dr. Morton, in a meeting in which cataphoresis was being discussed, stated that he had discovered a new means of producing anæsthesia with cocaine applied under pressure, but the experiment which he reported was not that kind of pressure which we now use,—namely, mechanical pressure. He dissolved the cocaine in guaiacol and ether and applied them under a cover-glass on his arm, and incisions with a lancet could be made on his arm painlessly, from which he gathered the idea that it was the chemical evaporation of the ether which caused a pressure forcing the cocaine into the tissues. Immediately after this a preparation was put upon the market called "Vapocain;" other dentists around the country applied mechanical pressure instead of this method.

I am intensely interested in the statement made by Dr. Ash that he used the unvulcanized rubber and cocaine over ten years ago. If this is correct, pressure anæsthesia was first done by Dr. Ash.

*Dr. Ash.*—I hope that none of the gentlemen will suppose that I make any claim for priority in the matter of pressure anæsthesia.

*Dr. M. I. Schamberg.*—It is well that attention be directed to the fact that we are blaming arsenic and cocaine pressure for conditions for which they, in a large proportion of cases, are not responsible. It is also well to remember that the removal of the pulp is a surgical procedure. Whenever a pulp is removed it is to be expected that some reaction will follow the severance of this once vital tissue from the remainder of the body. When arsenic is used, if it passes beyond the end of the tooth it will cause tissue destruction as it does in the pulp-chamber. When cocaine reaches the periapical tissues it temporarily anæsthetizes the tissues with which it comes in contact. It is a question in my mind whether cocaine so used is as likely to cause absolute destruction or permanent harm to the periapical tissues as is arsenic. Patients subjected to the cocaine pressure method occasionally show slight toxic symptoms, proving that the drug may enter the general circulation. The action of the arsenic, however, is purely local. The soreness following pulp extirpation is probably largely due to the traumatism attending this slight surgical procedure. If you remove a gangrenous finger or amputate any part of the body, even though it be not diseased, you must look for a certain amount of inflammatory reaction. You ought, therefore, to expect a certain amount of reaction following the removal of a pulp. It is, however, the permanent after-troubles which are of such serious moment to us when dealing with pulp-canals, and the cause of these, I believe, can readily be appreciated when one examines teeth that are removed with abscesses and other periapical troubles associated with them. In dealing with the pulp we work largely in the dark and without a clear perception of the minuteness, the length, and the tortuosity of the canals we treat. It is not surprising, then, that portions of the pulp remain to undergo putrefaction, and that fillings frequently fail to reach the ends of teeth. I maintain that we are blaming methods for the destruction of the pulp which are good and useful, and are overlooking the more potent etiological factors in the production of lame teeth. Dr. Osler, in his recent address at Johns Hopkins University, shocked the entire world by stating that man's time is up at forty, asserting the comparative uselessness of men above forty and advocating the enforced retirement of all men at sixty. It has occurred to me that Dr. Osler unwisely failed to provide for the chloroforming of the large number of men at twenty who are more worthless than many at sixty.

This principle applies equally well to the teeth of man. Inasmuch as modern dentistry teaches us to conserve the teeth for twenty, forty, or sixty years, we naturally, during these long periods, in consequence of our laudable attempts, encounter many more abscesses and other tooth-disturbances than did the practitioner of old. One of the previous speakers has stated that the old custom was to place arsenic in the tooth, allow it to remain, and upon the first indication of trouble the tooth was removed. Such a tooth, according to their judgment, had lost its field of usefulness. In our present endeavor to make a tooth useful for forty or sixty years we find ourselves occasionally confronted with conditions which could never have existed had the tooth been lost at twenty. In spite of what has been said, if all work upon the pulp and pulp-canal was done under strict surgical antisepsis, much of the trouble that we encounter with devitalized teeth would be averted.

*Dr. Goslee* (closing).—In the anticipation of my visit to this city, if there was one thing I could have wished for more than another, it would doubtless have been to have heard the expressions that my good friends have made to-night in support of the few things which I have said in my paper, because we in Chicago look up to the profession of Philadelphia with considerable deference, and if I had asked for one special thing more than another it would have been for the approval of Dr. Truman and Dr. Darby. The tribute paid me by Dr. Darby is highly appreciated. From his personal and professional life and from his writings I have received much inspiration.

It is true that we do have trouble in the use of arsenic, as we do in the application of cocaine and pressure. I called attention, however, to the fact that, as a rule, many do not discriminate in the application of arsenic, or between the size of the tooth and of the pulp, and that we often make no discrimination in the physical condition of the patient upon which we operate. I think that this discrimination, however, has to do very largely with the successful application of such an agent as arsenic. I also think, at the same time, that a great many of the bad results which we do have resulting from cocaine and pressure can be averted by previously thoroughly sterilizing the tract so that the likelihood of infecting the tissue in the apical region will be overcome.

I do not know whether the period during which Dr. Funk went about demonstrating the present method of pressure anæsthesia antedates that attributed to Dr. Morton or not. We in Chicago learned to believe that Dr. Funk was probably the first man to introduce the mechanical means now employed. I took up this method as soon as I learned of it, and in my experimental work in the college in the first six months we removed probably ninety per cent. of pulps by this method. We did have much subsequent trouble, and, though only of temporary nature, its use was for this reason almost entirely discontinued for a time. Since then we have begun to again use it more extensively, and in my personal practice I also use it to a large extent and perhaps always shall. I think that success with any method, however, depends largely upon the selection of the proper one for the individual case.

Again let me thank you for the courtesy extended to me.

Upon motion of Dr. Darby, a vote of thanks was extended to Dr. Goslee for his interesting paper.

Otto E. Inglis,
*Editor Academy of Stomatology.*

